# Hepatic Proliferation and Angiogenesis Markers Are Increased after Portal Deprivation in Rats: A Study of Molecular, Histological and Radiological Changes

**DOI:** 10.1371/journal.pone.0125493

**Published:** 2015-05-28

**Authors:** Florent Guérin, Mathilde Wagner, Antoine Liné, Magaly Zappa, Magali Fasseu, Valérie Paradis, Valérie Vilgrain, Bernard E. Van Beers, Josette Legagneux, Richard Moreau, Philippe Lettéron

**Affiliations:** 1 Department of Pediatric Surgery, Hôpitaux Universitaires Paris-Sud (Bicêtre), Assistance Publique Hôpitaux de Paris (AP-HP), Université Paris Sud-Paris11, Le Kremlin Bicêtre, France; 2 Institut National de la Santé et de la Recherche Médicale (INSERM): Unité Mixte de Recherche (UMR) 1149, Centre de Recherche sur l’Inflammation, Faculté de Médecine Xavier Bichat, Université Denis Diderot-Paris 7, Paris, France; 3 Laboratoire de Microchirurgie, Agence Générale des Equipements et Produits de Santé (AGEPS), Assistance Publique Hôpitaux de Paris (AP-HP), Paris, France; 4 Department of Radiology, Hôpitaux Universitaires Paris Nord Val de Seine (Beaujon), Assistance Publique Hôpitaux de Paris (AP-HP), Université Denis Diderot-Paris 7, Clichy, France; 5 Department of Pathology, Hôpitaux Universitaires Paris-Nord-Val-de-Seine (Beaujon), Assistance Publique Hôpitaux de Paris (AP-HP), Université Denis Diderot-Paris 7, Clichy, France; 6 Department of Hepatology, Hôpitaux Universitaires Paris-Nord-Val-de-Seine (Beaujon), Assistance Publique Hôpitaux de Paris (AP-HP), Université Denis Diderot-Paris 7, Clichy, France; University of Navarra School of Medicine and Center for Applied Medical Research (CIMA), SPAIN

## Abstract

**Background & Aims:**

To determine the pathogenesis of liver nodules, and lesions similar to obliterative portal venopathy, observed after portosystemic shunts or portal vein thrombosis in humans.

**Methods:**

We conducted an experimental study comparing portacaval shunt (PCS), total portal vein ligation (PVL), and sham (S) operated rats. Each group were either sacrificed at 6 weeks (early) or 6 months (late). Arterial liver perfusion was studied in vivo using CT, and histopathological changes were noted. Liver mRNA levels were quantified by RT-QPCR for markers of inflammation (*Il10*, *Tnfa*), proliferation (*Il6st*, *Mki67*, *Hgf*, *Hnf4a*), angiogenesis: (*Vegfa*, *Vegfr 1*, *2* and *3; Pgf*), oxidative stress (*Nos2*, and *3*, *Hif1a*), and fibrosis (*Tgfb*). PCS and PVL were compared to the S group.

**Results:**

Periportal fibrosis and arterial proliferation was observed in late PCS and PVL groups. CT imaging demonstrated increased arterial liver perfusion in the PCS group. RT-QPCR showed increased inflammatory markers in PCS and PVL early groups. *Tnfa* and *Il10* were increased in PCS and PVL late groups respectively. All proliferative markers increased in the PCS, and *Hnf4a* in the PVL early groups. *Mki67 and Hnf4a* were increased in the PCS late group. *Nos3* was increased in the early and late PCS groups, and *Hif1a* was decreased in the PVL groups. Markers of angiogenesis were all increased in the early PCS group, and *Vegfr3 and Pgf* in the late PCS group. Only *Vegfr3* was increased in the PVL groups. *Tgf* was increased in the PCS groups.

**Conclusions:**

Portal deprivation in rats induces a sustained increase in intrahepatic markers of inflammation, angiogenesis, proliferation, and fibrosis.

## Introduction

Various liver nodules and intrahepatic pathological changes have been observed in the human liver after both surgical and congenital portosystemic shunts, as well as extrahepatic portal vein thrombosis. These include benign liver tumours such as focal nodular hyperplasia and liver cell adenoma, and malignant tumours such as hepatocellular carcinoma and hepatoblastoma [1–4]. Commonly, quiescent histopathological periportal changes with similar appearances to obliterative portal venopathy have been observed, including paucity of the portal vein, perisinusoidal fibrosis, ductal proliferation, and an increase in the number of capillaries or arterioles in the portal space [2, 3, 5]. These changes have also been observed in animals with congenital or acquired portacaval shunt (PCS) [6, 7]. However, only one study has demonstrated liver nodules after PCS in rats, [8].

Recent experimental studies have focused on the metabolic and inflammatory changes following PCS in rats [9–11]. These studies however, focused on the extrahepatic expression of inflammatory molecules and liver atrophy, rather than intrahepatic changes. Currently, little is known regarding the intrahepatic expression of growth factors and markers of inflammation, angiogenesis, and fibrosis in the liver, following portal vein thrombosis or PCS. Expression of these growth factors might explain the paradox between liver atrophy, periportal vasculogenesis and fibrosis, and the abnormally high occurrence of nodules in humans.

The aim of this study was to assess the morphologic changes (radiological and histopathological), and the expression of markers of inflammation, angiogenesis and fibrosis in the liver, following experimental portal vein deprivation.

## Materials and Methods

### Animals

Experiments were conducted in 200–260g 6 week-old male Wistar rats: *ratus norvegicus* (Laboratoire Janvier, France). The experimental procedures used in this study complied with the principles and practices of the Ethical Guidelines from European Community Council Directive (2010/63/EU). Details of this study were approved by the local Bichat-Debré Ethics Committee in accordance with the French law for the protection of animals.

The condition of the animals was checked twice daily, and once daily over the week-end and bank holidays. Humane endpoints used for this experiment were based on clinical symptoms: no return to normal activity 48 hours after the procedure, lack of activity, aggressive behaviour, weight loss, and loss of appetite. If any of these endpoints were reached, analgesia with Buprenorphine (0.05mg/Kg/8h) was started followed, by planned euthanasia using Isoflurane overdose (10% 4l/min).

### Experimental design

The animals were divided (randomised block design) into three groups: rats with end-to-side portacaval shunt (PCS), complete portal vein ligation (PVL), and sham-operated rats (S). Each group was divided into two subgroups, early (6 weeks post-operatively) and late (6 months post-operatively) sacrifice groups. The initial study was designed with 13 animals in each early and late PCS and PVL group, expecting an arbitrary mortality rate of 20% in these groups, and 12 in each S group for an expected mortality rate of 10% in the S group. These expected mortality rate were approved by our institutional ethics committee. There were 9/26 (i.e. 34% CI [17–55]) deaths in the PCS groups, 7/26 (i.e. 27% CI [11–47]) in the PVL group, and 2/24 (i.e. 8% CI [1–27]) in the S group. The number of remaining animals in each group and subgroup is displayed in Table 1.

**Table 1:** Distribution of the remaining rats, available for experiments between PCS, PVL, and S groups in early and late sub-groups.

	PCS	PVL	S
**Early**	9	13	12
**Late**	8	6	10

### Surgical Procedures

The rats were anaesthetized with Isoflurane (2%, 2l/min). Intra-operative and post-operative analgesia was achieved with a subcutaneous injection of buprenorphine (0.05 mg/kg/8h for 48 hours). All groups had the same first steps of the procedure. With an operating microscope (OPMI-1FR, Carl Zeiss; Oberkochen, Germany) a median laparotomy was performed, and the intestines were retracted to expose the inferior vena cava and the portal vein. The end-to-side PCS was performed according to the technique previously described by Lee [12]. As the rats do not tolerate one-step total PVL, we performed the two-step procedure as described by Koshy [13]. After clamping the inferior vena cava (IVC) to reproduce the same condition as for the PCS, a 21 gauge metallic stent was placed along the portal trunk and stitched around the portal vein with 7/0 silk sutures. The stent was removed 30 minutes later, as well as the IVC clamp, producing portal stenosis. The second step consisted of a second laparotomy 48 hours later. The stenosed portal trunk was tied off to complete a portal vein ligation. The PVL and S groups underwent a general anaesthetic during the same period to expose them to the same amount of anaesthetics, thereby avoiding bias in our analysis.

Sham operated rats had both IVC and portal vein clamped for the same duration as the PCS and PVL groups. All the clamps were released after 30 minutes, and the abdominal wall was closed. The animals were recovered for 6 hours before returning to a normal diet and housing.

### Imaging

In-vivo perfusion computed tomography (CT) scans were performed before sacrifice. Following an intra-muscular anaesthetic using ketamine (35 mg/kg) and xylazine (7 mg/kg), a polyurethane catheter was placed in the femoral vein. A CT-scan was performed (VCT 64, General Electric, Milwaukee, WI, USA), using the following parameters: 80 kV, 160 mA, Field of view: 15 cm. Initially, an abdominal CT without contrast was performed. Subsequently, 0.1ml/100g of an iodinated contrast agent (Visipaque, 270mg/ml, GE Healthcare, Ireland) was manually injected, and 4 CT slices (thickness = 5 mm) centred on the liver hilum were acquired every 0.5 seconds over 1 minute, and every 4 seconds for 4 subsequent minutes.

**Fig 1:**
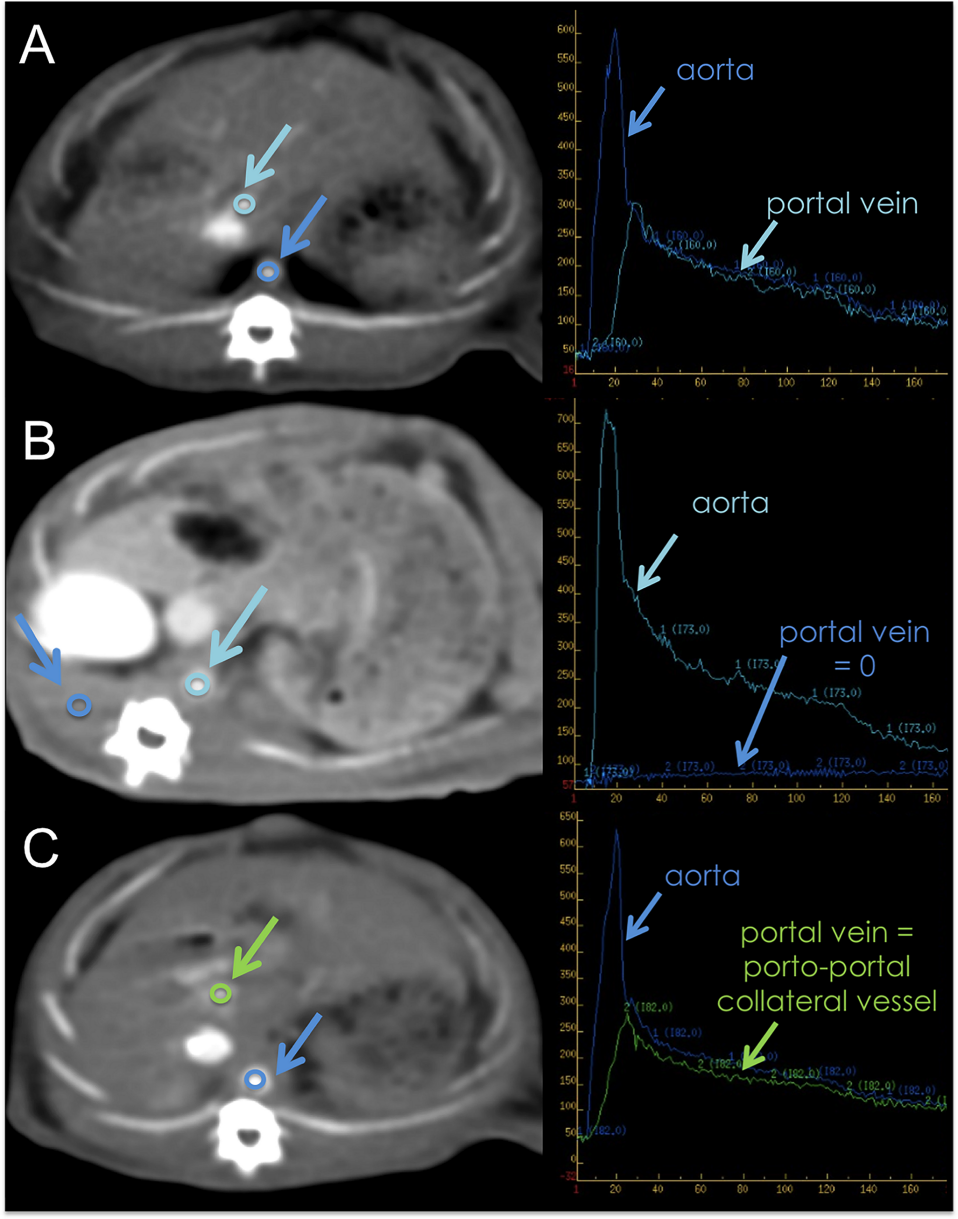
Methods for Perfusion CT scan. Perfusion CT scan (left) and time-density curves of vascular ROIs after contrast material injection (right). A: sham group; B: portocaval shunt; C: portal vein ligation. Note the portal cavernoma on rats with total portal vein ligation (Green arrow).

Radiological analysis: the CT images were transferred to a workstation (Advantage Windows, GE Healthcare, Buc, France) for perfusion analysis by a radiologist (MW). Two vascular regions of interest (ROI) were drawn: one in the aorta, and one in the portal vein for the S group, and in the largest collateral vessel in the PVL group. In the PCS group, a ROI was placed in the paravertebral muscle, as there was no portal blood flow. In all rats, a parenchymal ROI was located in the right median lobe of the liver, avoiding the large vessels (Fig 1). CT attenuation curves were used to calculate the blood perfusion (ml/min/100ml), blood volume (ml/100ml), and the arterial fraction (%) using the Johnson-Wilson distributed model [14] with the GE CT perfusion 4D software (General Electrics Healthcare, CT, USA). Total blood flow (ml/min) was calculated as blood perfusion times liver volume.

### Histological and molecular analysis

All animals were sacrificed following CT examination during the same anaesthesia. They were weighed before a midline abdominal incision was made, with subsequent exsanguination via the IVC. The liver was removed and weighed. The liver/animal weight ratio was calculated.

Samples of the right, middle and left lobes of the liver were stored in 10% formalin for standard histopathological studies with hematoxylin-eosin-safran staining. A qualitative analysis of the portal spaces was performed.

For molecular analysis, 50mg of each lobe were rapidly frozen in acetone, chilled with dry ice and stored at -80°C for messenger ribonucleic acid (mRNA) quantification by reverse transcriptase polymerase chain reaction amplification (RT-PCR). Total RNA was extracted from cells using Trizol reagent (Invitrogen, Cergy-Pontoise, France). The quality and integrity of RNA was evaluated using an Agilent 2100 bioanalyser (Agilent Technologies, Inc. Santa Clara, CA). One μg of total RNA was incubated in genomic Deoxyribonucleic acid (gDNA) Wipeout Buffer at 42°C for 2 minutes for gDNA contamination. After gDNA elimination, the RNA sample was ready for reverse transcription using a master mix prepared from Quantitect reverse transcription (Qiagen, Courtaboeuf, France). The differential expression of cytokine genes was assessed by real time quantitative polymerase chain reaction (RT-QPCR) using the SYBR Green based detection system on LightCycler 480 (Roche Diagnostics, Mannheim, Germany). *Gapdh* was used as housekeeping gene. The analysis of relative change in mRNA expression of target gene was based on 2-delta-delta-Ct method, therefore both PCS and PVL groups were compared to the value of the Sham which was 1, [15]. Genes of interest, displayed with their primers in Table 2, were classified as inflammation, proliferation, cell oxidative stress, angiogenesis, and fibrosis genes.

**Table 2:** Gene, protein and the corresponding primers used for RT-QPCR studies.

Category	Gene	Encoded protein	Upper primer	Lower primer
Inflammation	*Tnf Tnfa*	Tumour necrosis factor	5'GCCCAGGCAGTC AGATCATCTT3'	5'CCTCAGCTTGAGGGTTTG CTACA3'
Inflammation	*Il10*	Interleukin 10	5' GGC GCT GTC ATC GAT TTC TTC 3'	5' AGA TGC CTT TCT CTT GGA GCT TAT T 3'
Proliferation	*Hgf*	Hepatocyte growth factor	5’TGTTTTGTTTTGGCACAGGA3’	5’TCGTTCCTTGGGATTATTGC3’
Proliferation	*Il6st*	Interleukin-6 signal transducer	5’GCACGACTATGGCTTCGATT3’	5’CACAGAAGAAGGTGGGAAGG3’
Proliferation	*Mki67*	Antigen KI-67	5’TCACTTTTCTGGTGACTTCTTGTT3’	5’GGCCAAGAAAGATGCAAAAA3’
Proliferation	*Wnt2*	Wingless-type MMTV integration site family member 2	5’GGGAAGTCAAGTTGCACACA3’	5’GAAGCCAACGAAAAATGACC3’
Proliferation	*Hnf4a*	Hepatocyte nuclear factor 4-alpha	5’CGGCCTTCTGTGAACTTCTT3’	5’GAGCAGCACATCCTTGAACA3’
Cellular stress	*Nos2*	Nitric oxide synthase. inductible	5’GAACTGGGGGAAACCATTTT3’	5’GGTGCAGAAGCACAAAGTCA3’
Cellular stress	*Nos3*	Nitric oxide synthase.endothelial	5’GAGGGGAGCTGTTGTAGGG3’	5’AGCATGAGGCCTTGGTATTG3’
Cellular stress	*Hif1a*	Hypoxia inducible factor 1, alpha subunit	5’GCAACGTGGAAGGTGCTG 3’	5’CGTCATAGGCGGTTTCTTGTAG3’
Angiogenesis	*Vegfa*	vascular endothelial factor A	5’GACGTCCATGAACTTCACCA3’	5’GTACCTCCACCATGCCAAGT3’
Angiogenesis	*Flt1 VEGFR-1*	FMS-related tyrosine kinase 1 Vascular endothelial growth factor receptor 1	5’TTGGTCTCAGTCCAGGTGAA3’	5’GAGGAGCTTTCACCAAATGC3’
Angiogenesis	*Kdr Vegfr-2*	kinase insert domain protein receptor Vascular endothelial growth factor receptor 2	5’CGGCCTTCTGTGAACTTCTT3’	5’GAGCAGCACATCCTTGAACA3’
Angiogenesis	*Flt4 Vegfr3*	Fms-related tyrosine kinase 4 Vascular endothelial growth factor receptor 3	5’CCCAGTCACTGCCTTTCTGT3’	5’AAAGCCCTTCATCAGTGTCG3’
Angiogenesis	*Pgf Plgf*	Placenta growth factor	5’TTCCTCTTCCCCTTGGTTTT3’	5’GGGATCCACATTCCTACGTG3’
Fibrosis	*Tgfb Tgfb1*	Transforming growth factor beta-1	5’ACTTCCAACCCAGGTCCTTC3’	5’GGAGAGCCCTGGATACCAAC3’

Western blots were used to measure the protein expression of Hif1a in the liver.

In tissue samples (50–60 mg), nuclear and cytoplasmic fraction protein concentrations were determined by the BCA assay. Total protein equivalents (50 μg) for each sample were separated by by Nupage 4–12% tris gel (life technologies, Ca, USA) and were transferred onto nitrocellulose membrane. The membrane was immediately placed into blocking buffer containing 5% non-fat milk in TBS and 0.01% Tween-20 at 20°C for 1 h. The membrane was incubated with rabbit polyclonal Hif1a (1:1000) overnight at 4°C, followed by incubation in an anti-rabbit IgG-horseradish peroxidase conjugated antibody (1:10 000). The membranes were incubated with ECL plus detection reagents (Amersham LifeScienceInc, Buckinghamshire, UK), exposed to FUSION-FX Chemiluminescence System (BioRad, Nanterre, France). Pre-stained protein markers were used for molecular weight determinations.

### Statistics

Statistical analysis was performed using GraphPad Prism software (Graphpad Inc., Ca, USA). The results were expressed as median and range. The 3 groups were analysed using Kruskal-Wallis or Mann Whitney tests with Dunn’s multiple comparison test. Correlations were performed using the Spearman test. Results were considered significant when P < 0.05

## Results

### Early sacrifice group (6 weeks)

Mortality: There were 4 deaths in the early PCS group (1 intra-operative cardiac arrest during the clamping procedure, 1 during the CT scan secondary to bleeding at the catheter insertion site, and 2 late sudden deaths of unknown cause (at 4 and 5 weeks following the procedure), despite daily check of humane endpoints, and post mortem examination demonstrating no evidence of shunt thrombosis.

The liver-weight ratio was significantly lower in the PCS group (2.21% [2.11–2.37]) than in PVL (3.33% [3.17–3.40] (P < 0.001)) and S groups (3.45% [3.29–3.67] (P < 0.001)).There was no statistical difference between PVL and S (P = 0.089). Histopathological features in the liver were similar in PCS and PVL, with no changes in the S group.

**Fig 2:**
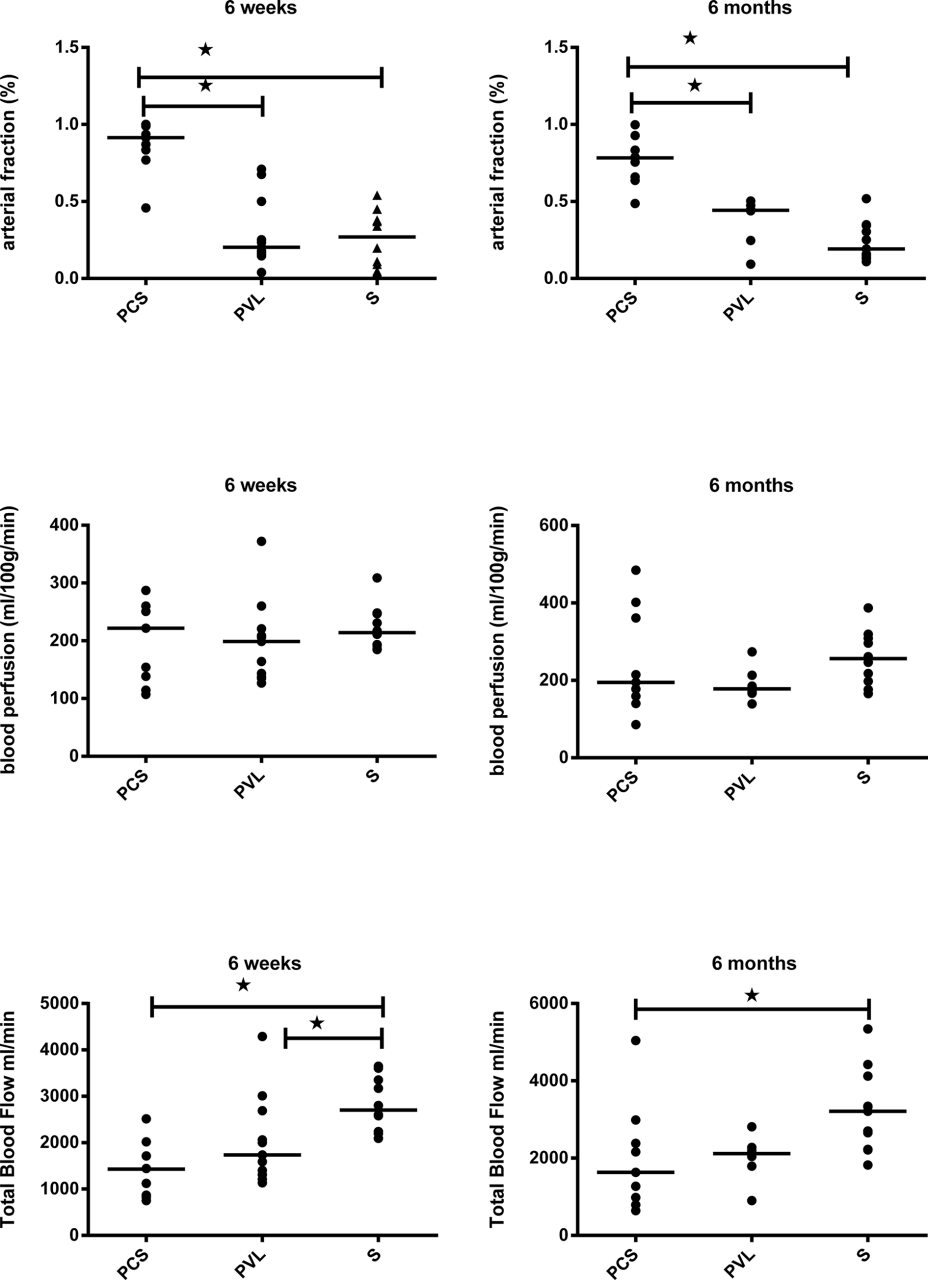
Results of imaging evaluation of the three experimental groups. Aligned dot plots with bar at median for the expression of hepatic arterial fraction,blood perfusion, and total blood flow at 6 weeks and 6 months. Hepatic arterial fraction is significantly increased in the rats with portacaval shunts. At 6 weeks and 6 months, and total blood flow is decreased in both PCS and PVL at6 months compared to Shams. PCS: Portacaval shunt. PVL: Portal vein ligation. S: Sham. *: significant difference.

CT demonstrated the development of a portal cavernoma in all rats in the PVL group (13/13), enabling identification of the portal ROI in the largest collateral vessel (Fig 1). Arterial fraction was 0.86 [0.46–1] in the PCS group, 0.30 [0.04–0.71] in the PVL group, and 0.26 [0.04–0.54] in the S group. The arterial fraction was significantly higher in the PCS than in the S group (P = 0.016), but did not differ significantly between the PVL and S groups (P = 0.151). Blood perfusion and total blood flow did not differ significantly between the three groups (P = 0.181 and P = 0.145, respectively) (Fig 2).

**Fig 3:**
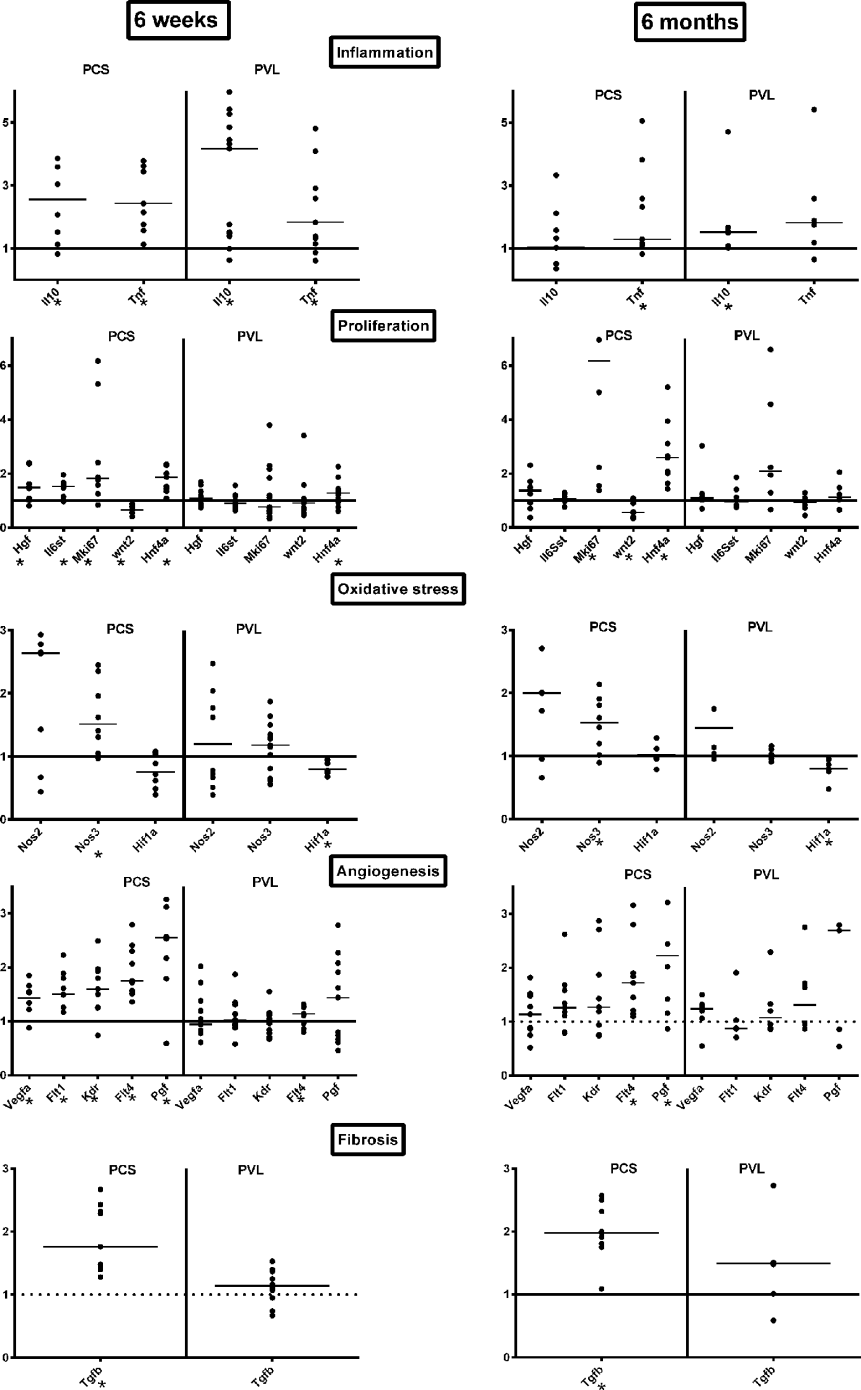
Results of mRNA quantification by RT-QPCR. Aligned dot plots with bars at median for the expression of inflammatory (*Tnfa*, *Il10*), proliferative (*Hgf*, *Il6st*, *Mki67*, *Wnt2*, *Hnf4a*), Oxidative stress (*Nos2&3*), angiogenetics (*Vegfa*, *Flt1/VEGFR-1*, *Kdr/Vegfr2*,*Flt4/Vegfr3*), and fibrotic (*Tgfb*) mRNA markers by RT-QPCR according to the Delta-Delta Method (Sham = 1) at 6 weeks and 6 months. PCS: Portacaval shunt. PVL Portal vein ligation. S: Sham. *: significant difference.

Molecular studies using RT-QPCR showed a significant increase of *Tnfa* in both PCS and PVL, compared to S (respectively P = 0.010 and P < 0.003). This was counterbalanced by an increase in the anti-inflammatory molecule *Il10* in both PCS and PVL compared to S (respectively P = 0.023; P = 0.001). The proliferative markers *Hgf*, *IL6st*, *Mki67*, *Hnf4a* were increased in the PCS group compared to the S group (P = 0.034; P = 0.023; P = 0.015; and P = 0.003), whereas only *Hnf4a* was significantly increased in the PVL group (P = 0.044). Wnt2 was significantly decreased compared to S group both in the PCS group (P = 0.015), but not in the PVL group (P = 0.269). The cellular stress marker *Nos3* was increased only in the PCS (P = 0.015) but not in the PVL group (P = 0.313). *Hif1a* was not elevated in the PCS group, but significantly decreased in the PVL group (P = 0.03). Angiogenesis markers *Vegfa*, *Flt1 (VEGFR-1)*, *Kdr (Vegfr-2) Flt4 (Vegfr3);* and *Pgf* were significantly increased in the PCS group, compared to the S group P = 0.015; P = 0.003, P = 0.015; P = 0.003, and P = 0.015 respectively, whereas only *Flt4* was increased in the PVL group (P = 0.042). Fibrosis marker *Tgfb* was increased only in the PCS group (P < 0.003); (Fig 3).

### Late sacrifice group (6 months)

Mortality: There were 5 deaths in the late PCS group (2 intra-operative cardiac arrests during clamping, 3 late sudden deaths of unknown cause (at 5, 9 and 12 weeks post-procedure without evidence of shunt thrombosis on post mortem examination). There were 7 deaths in the late PVL group (2 intra-operative cardiac arrests, 1 during CT scanning, 1 secondary to bleeding from the catheter insertion site, and 3 late sudden deaths of unknown cause (at 6, 7, 13 weeks post procedure despite no evidence of shunt thrombosis on post-mortem examination), and 2 late sudden deaths of unknown cause in the S group (4 and 7 weeks post-procedure), despite no evidence of shunt thrombosis on post mortem examination.

All the late deaths in this study were not detected by the humane endpoints methods. The liver weight ratio was significantly lower in the PCS group: 2.11% [2.00–2.31] than in the PVL group 2.46% [2.35–2.72] (P = 0.004), and the S group 2.73% [2.62–3.00] (P < 0.001).

**Fig 4:**
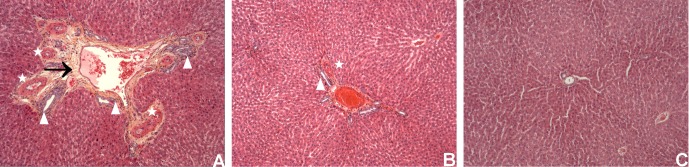
Liver biopsies at 6 months. View centered on the portal space, with HES staining, X20 enhancement. Rats with portacaval shunt (A), portal vein ligation (B), and Sham operated (C).

Histopathological changes observed included periportal fibrosis, and ductal and capillary proliferation, with more than two arteries in more than 30% of portal spaces for PCS and PVL. The only difference between PCS and PVL was portal vein congestion in the PVL group (Fig 4). These changes were similar to the observed portal space changes following portacaval shunts in humans [2].

On Fig 4A, there are multiples arteries (stars) with ductular proliferation (white arrowheads), and fibrosis (black arrow). The same pattern with less fibrosis is seen on the liver biopsy of a rat with PVL (B). (C) shows a normal portal space on a sham rat.

As demonstrated in the early group, all the rats (6/6) in the PVL group had radiological evidence of development of a portal cavernoma (Fig 1). The arterial fraction was 0.76 [0.49–1] in PCS group, 0.37 [0.09–0.50] in the PVL group, and 0.24 [0.11–0.52] in the S group. The arterial fraction was significantly increased in the PCS compared to the S group (P < 0.001), but did not differ significantly between the PVL and S groups (P = 0.315). Blood perfusion was the same in all three groups (P = 0.326), but total blood flow was significantly lower in both the PCS: 1.98 ml/min [0.64–5.04] and PVL group: 2.00 ml/min [0.90–2.81], than in the S group: 3.21 ml/min [1.82–5.34] (P = 0.022 and P = 0.025 respectively) (Fig 2).

Molecular studies using RT-QPCR showed a significant increase of *Tnfa* only in the PCS group compared to S group (P = 0.027), whereas *Il10* was increased only in the PVL group (P = 0.031). Proliferative markers *Mki67* and *Hnf4a* were increased only in the PCS group compared to the S group (P = 0.003, P = 0.003 respectively). *Wnt2* was significantly decreased only in the PCS compared to the S group (P = 0.031). *Nos3* remained higher in the PCS group (P = 0.023) and *Hif1a* significantly decreased in the PVL group (P = 0.03). Among the markers of angiogenesis, only *Flt4 (Vegfr3)* and *Pgf* remained significantly increased in the PCS group, compared to the S group (P = 0.003; P = 0.015 respectively), whereas none was increased in the PVL group. The marker of fibrosis, *Tgfb*, was increased only in the PCS group compared to S (P < = 0.003) (Fig 3).

In the early group, there was a significant correlation between the arterial fraction in the PCS and PVL group, with *Hnf4a* (r = 0.46, P = 0.045), and *Flt4 (Vegfr3)* (r = 0.69, P < 0.001). In the late group, there was a significant correlation between the arterial fraction of PCS and PVL with *Hnf4a* (r = 0.60; P = 0.019), *Nos3* (r = 0.61; P = 0.017), *Flt4 (Vegfr3)* (r = 0.60; P = 0.019) and *Tgfb* (r = 0.53; P = 0.003).

**Fig 5:**
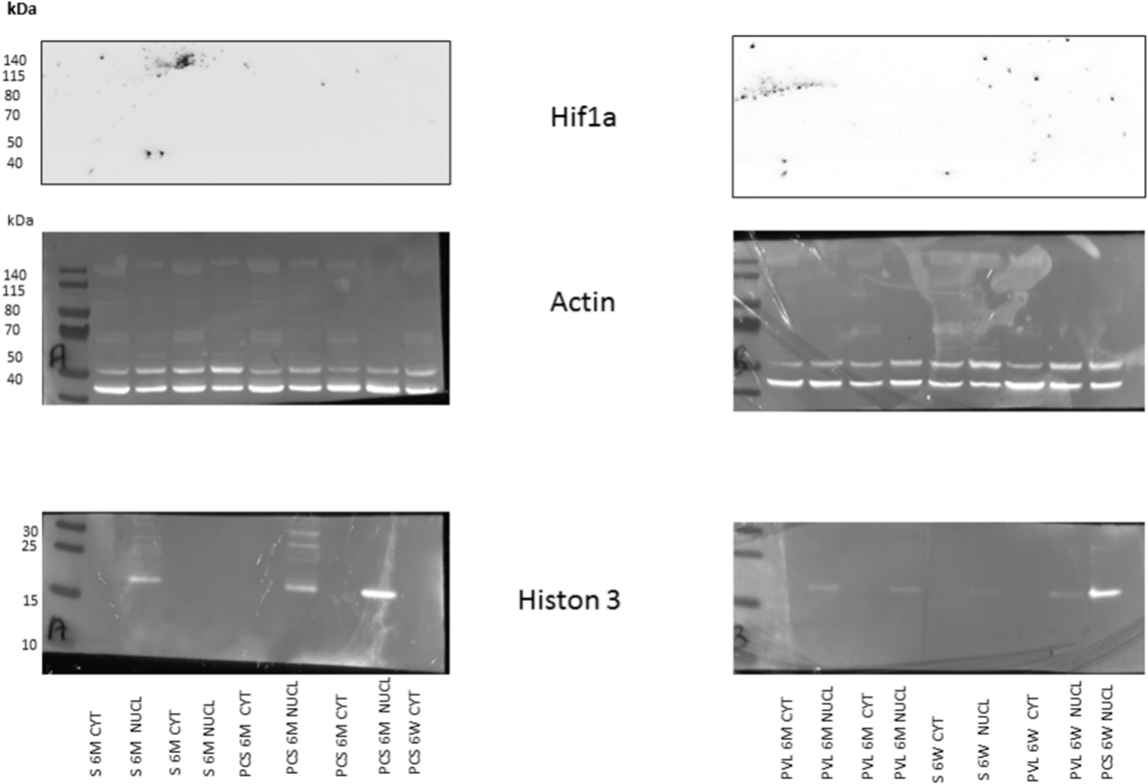
Western blot of *Hif1a* protein in the liver at 6 weeks (6W) and 6 Months (6M) in Sham (S), Portacaval shunt (PCS), and Portal vein Ligation (PVL) rats. Nuclear (NUCL) and cytoplasmic (CYT) protein were fractioned and assayed for Histon 3 to serve as a nuclear marker and Actin to serve as a protein marker. There is no staining for Hif1a in each 6 weeks and 6 months groups.

Hif1a western blot were negative at 6 Weeks and 6 months for both PCS and PVL groups (Fig 5).

## Discussion

In humans, portal deprivation resulting from PCS or portal vein thrombosis, has been observed to induce a wide spectrum of liver nodules [1–4, 16]. Portal deprivation is also known to induce fibrosis and arterial proliferation in the portal space [2, 5, 17]. The underlying mechanisms of these changes however, remain unclear. Our experimental study has demonstrated that portal deprivation in rats induces sustained intrahepatic expression of inflammatory, angiogenic, proliferative, oxidative and fibrogenic molecules, despite liver atrophy. These changes occurred with a distinct pattern of expression, depending on whether the portal flow is minimal but present (PVL), or fully diverted from the liver (PCS).

Previous experimental studies have addressed the paradox between atrophy and the regenerative pattern observed in the liver and other organs after PCS [9, 10]. *Tnfa* is a pro-inflammatory and *Il10* an anti-inflammatory cytokine. Our results are different to those of Garcia et al. who reported no increase of *Tnfa* and *Il10* mRNAs in the liver in PCS rats compared to their control rats at one month, [9]. In our study, there was a sustained *Tnfa* increase in PCS compared to S, whereas *Il10* increased in the liver only in the early PCS group. We do not have a good explanation for this discrepancy apart from an earlier sacrifice time in their study (4 weeks). Our study demonstrated in both PCS and PVL groups an early, balanced pro and anti-inflammatory response, mediated by *Tnfa* and *Il10* respectively. At 6 months, *Il10* expression persisted in the PCS group, where portal inflow is patent via the cavernoma, demonstrated on CT imaging. This might support the idea that portal flow in the liver has an anti-inflammatory effect. All but *Wnt2* proliferative mRNA markers tested increased in the early PCS group, probably in response to injury caused by portal deprivation. The only proliferative marker found to be increased in the PVL group was *Hnf4a*. At 6 months, the proliferative markers *Mki67* and *Hnf4a* were present only in the PCS group. *Hnf4a* is a central transcriptional regulator of hepatocyte differentiation, and is also known to attenuate hepatic fibrosis induced by portal impairment in our PCS group [18, 19]. *Wnt2* which was decreased in the early and late PCS groups, is a protein which activates the *beta-catenin* pathway. The *Wnt2/beta-catenin* pathway is involved in liver organogenesis, differentiation, proliferation, but also tumorogenesis, but was never assayed in PCS models [20]. In our study, the decrease of *Wnt2* expression could be the result of downregulation caused by other pathways like *Hgf/c-Met*.

Among the nitric oxide synthases, only the *Nos3* (endothelial) was significantly increased in both early and late PCS groups. *Nos3* is expressed in endothelial cells and hepatocytes, and its over expression could reflect either the protective effects due to ischaemic changes, but also hepatocyte proliferation and regeneration following chronic liver injury secondary to PCS [21]. This result is different from the study by Garcia et al. in which *Nos2* (inductible) was increased in the liver without expression of *Nos3* [9]. We do not have any explanation for this discrepancy, except statistical bias for *Nos2* (our P value almost reached statistical significance at 6 weeks and 6 months, respectively P = 0.054 and P = 0.078). The fact that *Hif1a* mRNA and protein expression was not increased in the PCS groups and decreased in the PVL groups, shows that this transcriptional factor was not involved or down regulated in PCS or PVL livers. This could be related to the known interaction between *Hif1a* and *Wnt2* in ischemia reperfusion injury repair. The difference in *Hif1a* mRNA expression between the PVL and PCS groups could also explain the observed difference in pro-angiogenic (*Vegfa*) and fibrotic markers (*Tgfb*) [22].

The mRNAs of vascular endothelial growth factor A and its receptors *Flt1 (VEGFR-1)*, *Kdr (Vegfr-2) and Flt4 (Vegfr3)* were all increased in the early PCS group, before returning to basal levels in the late group, apart from *Vegfr3*. Similar results for *Vegfr-2* over-expression were found in spontaneous PCS in dogs [23]. *Vegfr-2* is the key receptor of *Vegfa* and induces NO synthesis via *Nos3*. This could also explain why *Nos3* was over-expressed in our study [24]. However, this is not a persistent effect, and other molecules such as *Pgf*, secreted by sinusoidal endothelial cells to promote vasodilatation, proliferation, and fibrosis [25, 26], might take over the *Vegfa/Vegfr-2* pathway.

Transforming growth factor-beta (*Tgfb*) is the most potent hepatic profibrogenic cytokine that also exerts its biological effects on tissue and organ development, cellular proliferation, differentiation, survival and apoptosis [27,28]. In our study, the increase in *Tgfb* expression may reflect the response to fibrosis, but also long-term hepatocyte proliferation. This may explain the occurrence of liver cell tumours in humans after PCS.

The results of the mRNA studies should be interpreted with caution, as we have not assessed most of the protein expression in liver parenchyma. In both PCS and PVL groups, we had no radiological or histopathological evidence of any liver nodules. Until now, only one previous study has reported liver nodules at 6, 12, and 18 months following PCS in rats [8]. This observation has never subsequently been demonstrated [7, 10, 29, 30]. Our study demonstrated an increase in growth factors, proliferation and angiogenesis, which may favour the growth of a pre-existing tumour rather than driving hepatocarcinogenesis itself. In this study, imaging was used to assess the different patterns of liver perfusion in PCS, PVL, and S. In PCS, the arterial fraction increased significantly to balance the lack of portal perfusion. However, overall total blood flow decreased along with the liver weight in order to maintain a liver perfusion. Similar trends were observed in the rats with portal vein ligation at 6 months, which may be attributed to the small number of animals in this group (Fig 2 **).** Increases in hepatic arterial fraction were observed to balance a decreased portal venous flow in both cirrhotic and normal livers in a study by Zipprich et al [31]. The observed changes in liver perfusion and volume in PCS and PVL are explained by the liver arterial versus portal balance. Following total or partial portal vein obstruction, the arterial fraction of perfusion increases, as part of the hepatic artery buffer response to maintain total liver blood flow [32–34]. When the hepatic artery buffer response capacity is reached, the total liver blood flow decreases and liver atrophy occurs, in order to maintain an intact blood liver perfusion [35, 36]. These observations were confirmed in our study, which demonstrated a decreased liver weight ratio along with maintenance in liver perfusion quantified non-invasively using perfusion CT.

Interestingly, the increase in arterial fraction was confirmed both at a macroscopic level on histopathology specimen, and by molecular biology studies. Correlations between arterial fraction and proliferation (*Hnf4a)*, angiogenesis (*VEGFR1*, *Vegfr2*, and *3*), and *Tgfb* expression were observed, suggesting that perfusion CT might be useful in monitoring arterial and fibrotic changes induced by portal deprivation in the liver, at a macroscopic scale.

The role of perfusion CT in clinical practise, especially for monitoring treatment in children, is limited due to radiation exposure. Perfusion MR imaging may therefore provide an alternative.

In conclusion, this experimental study has demonstrated the long-term effect of portal deprivation on mRNA expression of proliferative and angiogenic factors in the liver, suggesting that these changes are the result of portal vein deprivation, rather than being associated with the portal vein malformation. Pharmacologic intervention with the anti-angiogenic Sorafenib has already been performed in partial PVL rats in the past [37]. This molecule could be tested in the future, in both total PVL and PCS rats, and may reveal either its positive or negative effects on the markers already tested in this present study.
